# Metronomic cyclophosphamide and metformin inhibited tumor growth and repopulated tumor-infiltrating lymphocytes in an experimental carcinoma model

**DOI:** 10.1186/s13104-023-06651-1

**Published:** 2024-01-02

**Authors:** Heba Mohamed Zaki, Khadiga Mohamed Ali, Mona Younis Youssef Abd Allah, Amoura Mohamed Abouelnaga, Mohamed Elsaid Abdraboh, Osama Hussein

**Affiliations:** 1 Zoology Department, Faculty of Science, Mansoura University, Mansoura, 35516 Egypt; 2 Pathology Department, Faculty of Medicine, Mansoura University, Mansoura, 35516 Egypt; 3 Surgical Oncology Department, Faculty of Medicine, Mansoura University Oncology Center, Mansoura University, Mansoura, 35516 Egypt

**Keywords:** Metformin, Metronomic therapy, Tumor infiltrating lymphocytes, Animal model

## Abstract

Metformin is a widely used antidiabetic biguanide. Retrospective data demonstrated the association of metformin use with survival benefit in multiple tumor types. Interest in repurposing metformin to treat cancer has not been translated into encouraging clinical benefit. In animal models, metformin activated cytotoxic T cells and exerted an immune-mediated anticancer effect. The current research was conducted to investigate the possible therapeutic benefit of metformin in combination with metronomic cyclophosphamide in an experimental cancer model. Ehrlich ascites carcinoma was injected into the subcutaneous tissue to induce solid tumors in syngeneic mice. Exponential solid tumor growth ensued and was effectively arrested with the administration of a cytotoxic dose of parenteral cyclophosphamide. Alternatively, oral metformin and continuous, low-dose cyclophosphamide significantly inhibited tumor growth relative to untreated mice. The drug combination was well tolerated. Histopathological examination of the tumor showed an increased number of tumor-infiltrating lymphocytes and enhanced expression of granzyme B by this drug combination. The current data suggests a potential role of metformin and metronomic chemotherapy that warrants further investigation.

## Introduction

Interest in repurposing metformin to treat cancer has followed the impressive benefit observed in multiple epidemiological studies [1–8]. Several mechanistic theories explain the antitumor effect of metformin, including direct antiproliferative effects on cancer cells, direct and systemic metabolic actions, and immune-mediated mechanisms [9, 10]. Following the demonstration of the stimulatory effect of metformin on cytotoxic T cells in animal tumor models [11–13] and clinical studies [14], several experimental strategies were investigated with variable success, including combining metformin with radiotherapy [15], tumor vaccination [16], checkpoint blockade [17, 18], and cytotoxic chemotherapy [19].

Metronomic (continuous, low-dose) chemotherapy is a treatment modality that has a well-known immune-stimulatory effect in both experimental [20] and clinical [21, 22] settings.

This study was undertaken to explore the possibility of the therapeutic benefit of combined metformin and metronomic cyclophosphamide therapy.

## Methods

### Experimental model

Ehrlich ascites carcinoma (EAC) in female Swiss albino mice harboring 8–10-day-old ascitic tumors was purchased from the Egyptian National Cancer Institute, Cairo University. To induce solid tumor formation, EAC cells (2.5 × 10^6^) were injected subcutaneously in the right flank of six- to eight-week-old female Swiss albino mice weighing 20–25 gm purchased from Vacsera Co., Egypt.

Mice that developed palpable tumors were randomly assigned to five groups of five mice each. Four treatment groups and one untreated control group. Treatment was either cytotoxic (bolus, high-dose) cyclophosphamide (Cyclo), metronomic (continuous, low-dose) cyclophosphamide, metformin, or combined metformin and metronomic cyclophosphamide.

### Drugs

High-dose cyclophosphamide (Endoxan®, Baxter, CA^®^) was administered at a dose of 100–150 mg/kg by intraperitoneal injection every other day for 3 weeks followed by 2 weeks of rest.

Metronomic cyclophosphamide was described previously [23]. The drug was dissolved in distilled water (stock solution 20 mg/ml) and kept at 4 °C in the dark for one week. A total of 1.25 ml of stock solution was dissolved in 200 ml water at a concentration of 20 mg/kg dose.

The metformin “Glucophage^®^, Merck, NJ” dosage was 5 mg/ml dissolved in drinking water to provide a dose of 5 mg/kg. Drinking water was changed twice weekly.

### Physical parameters

Tumor volume was measured at baseline and weekly thereafter for 4 weeks. The tumor volume of each animal was calculated using the ellipsoid formula: (Volume = length x width^2^ × 0.5). At the end of the fourth week, all mice were euthanized by thiopental overdose (150 mg/kg) intraperitoneal injection, followed by cervical dislocation, and the tumors were resected, measured, and preserved in formalin.

### Histology and immunohistochemistry

Tumor-infiltrating lymphocytes (TILs) were assessed in H&E sections by counting all mononuclear cells in the peritumoral stroma. Tumor paraffin sections were deparaffinized and retrieved using 0.01 M citrate buffer (pH = 6). After washing with PBS, the sections were quenched with 3% hydrogen peroxide, washed, blocked with horse normal serum, and then incubated with primary antibodies against Granzyme B (Cell Signaling Technology, MA; Catalog # 44153) and PD-1/CD279 (Chongqing Biospes, China; Catalog # YPA1638) at 4 °C overnight. IHC scoring was performed for granzyme B according to Kavalar et al. [24]. In brief, lymphocytes with a sparsely granulated pattern were counted in three fields (x400) at highly cellular areas of the tumor. Reactivity was graded as mild, moderate or marked if < 5, 6–10 or ≥ 11 lymphocytes were present, respectively.

For PD-1 immune staining, the score used depended on combining the staining intensity with the percentage of immunoreactive cells. The staining intensity was scored from 0 (negative) to 3 (intense). The percentage of immunoreactive cells was scored as 1 (< 5% positive cells) or 2 (≥ 5% positive cells). Then, the immunoreactive score (IRS) was calculated by multiplying the staining intensity score by the score of the percentage of immunoreactive cells. In all immunohistochemistry (IHC) experiments, a blinded pathologist examined two independent specimens per condition.

### Statistical analysis

The results are presented as the median and interquartile range (IQR). The Mann‒Whitney test was used to compare normalized tumor volumes. Grubb’s test was used to detect outliers. JASP 0.17.1.0 (® University of Amsterdam) and GraphPad™ Quick Calculator were used for calculations.

## Results

### Combined therapy inhibited tumor growth

**Fig. 1:**
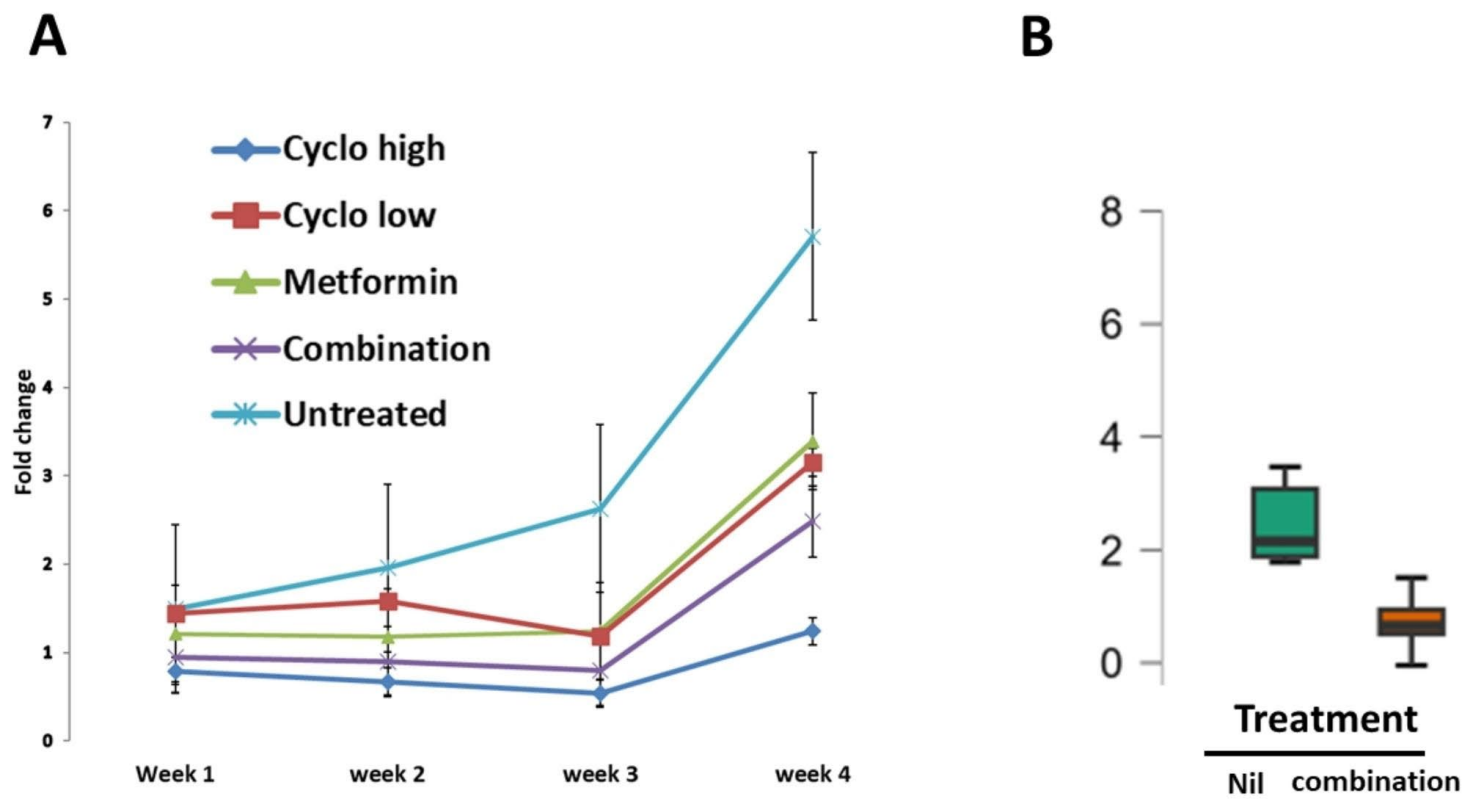
**A**) Change in tumor volume normalized to the original size at the start of treatment. **B**) volume at the third week in mice treated with combination of oral metformin and metronomic therapy compared to untreated tumors

**Fig. 2:**
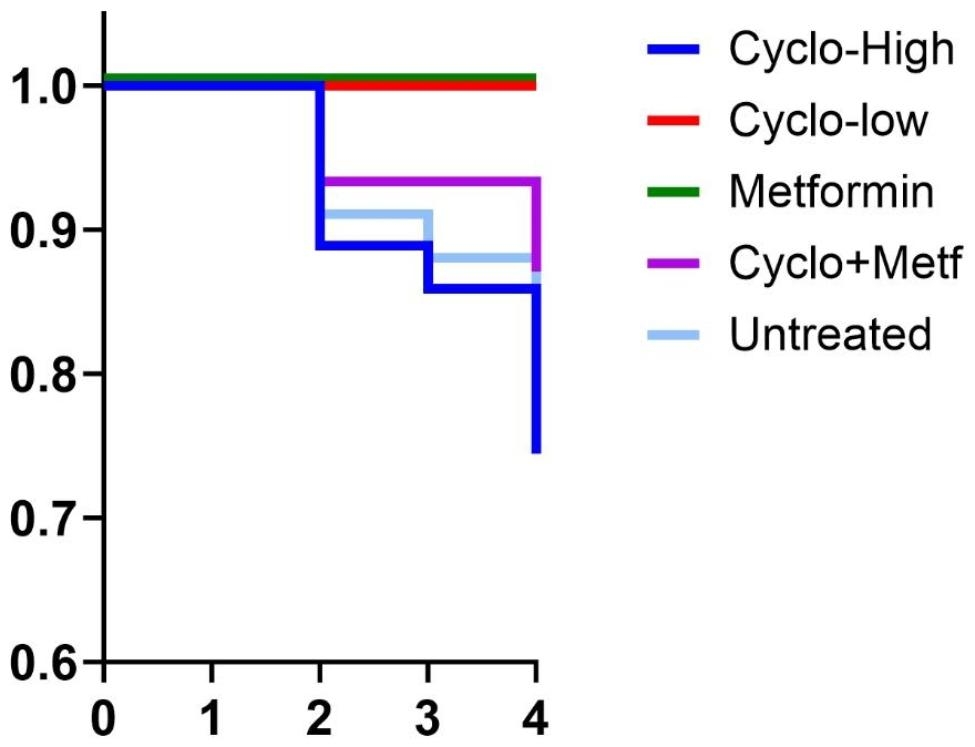
Kaplan-Meier’s curve of three pooled experiments showing percent survival at weeks three and four

Data from three independent experiments were pooled and normalized. The change in tumor volume relative to the baseline volume at the start of therapy was plotted over time. In total, 56 mice had measurable tumors in the fourth week. One mouse had an outlier measurement by Grubb’s test (value = 33.2, Z score = 3.013, P < 0.05) and was excluded. Mice treated with high-dose Cyclo showed arrested tumor growth. However, survival was shortened in eight mice. Combined metronomic therapy and metformin, but not either drug alone, significantly inhibited tumor growth compared with the untreated group (Fig. 1a). Growth inhibition was significant at week 3 (P = 0.021, Mann‒Whitney) and marginally significant at week 4 (P = 0.064) (Fig. 1b). At week 3, all mice treated with either metformin or low-dose cyclophosphamide alone were alive. At this time point, 80% of mice treated with combination therapy, 60% of those treated with high-dose chemotherapy, and 67% of untreated mice were alive (Fig. 2).

### Metformin repopulated tumor-infiltrating lymphocytes

Assessment of paraffin sections of untreated tumors showed inconsistent mild TIL infiltration. In this model, high-dose Cyclo abrogated the TIL population. Metformin and metronomic Cyclo alone or in combination consistently repopulated TILs (Table 1).

**Table 1:** Scoring of tumor-infiltrating lymphocytes (TILs). (Cyclo = cyclophosphamide, GZM B = granzyme B by immunohistochemistry). PD-1 score was calculated by multiplying the staining intensity score with the score of percentage of the immunoreactive cells

Specimen	TILs	GZM B	PD-1 score
Cyclo high	-	-	2
Cyclo high	-	-	
Cyclo + metformin	+	Mild	6
Cyclo + metformin	+	Mild	
Metformin	+	-	3
Metformin	+	Mild	
Cyclo low	-	-	4
Cyclo low	-	-	
Untreated	-	-	2
Untreated	+	Mild	

### Combination therapy induced granzyme B expression

**Fig. 3:**
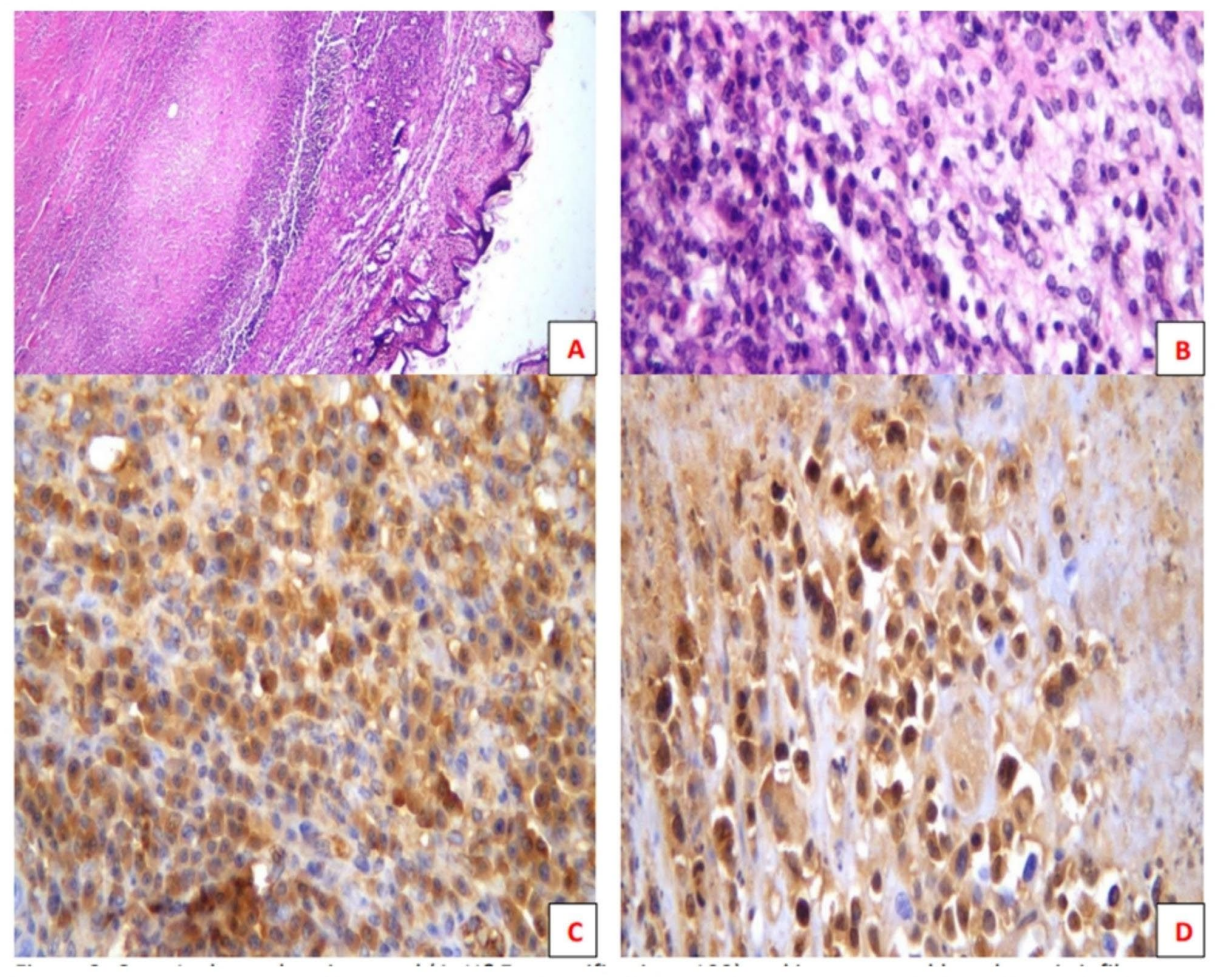
Peritumoral (**A**, H&E, magnification x100) and intratumoral lymphocytic infiltrate (**B**, H&E, magnification x400). Immunohistochemistry for granzyme b showed positive cytoplasmic reaction in both peritumoral (**C**) and intratumoral (**D**) lymphocytic infiltrate (**C** & **D**, IHC, magnification x400)

In general, the pattern of granzyme B expression followed the distribution of TILs in H&E sections. Consistent expression was observed in mice treated with combination therapy (Fig. 3).

### The effect of combination therapy was not attributable to lymphocyte rejuvenation

Contrary to the hypothetical assumption, PD-1 expression was elevated with metformin, metronomic Cyclo or their combination (Table 1).

## Discussion

In this study, an experimental murine cancer model was validated for therapeutic testing. High-dose Cyclo exerted constant arrest of otherwise exponential tumor growth. The therapeutic benefit of the metformin and metronomic Cyclo combination was demonstrated. Combination therapy but not monotherapy with either drug resulted in significant growth inhibition relative to untreated tumors. This growth inhibition came at the expense of statistically insignificant decrease in mice survival with combination therapy. With high-dose chemotherapy, the loss of mice even exceeded that of the untreated group.

Drug treatment was associated with recovery of the TIL population, and granzyme B expression was maximal in mice that received the combination therapy. Although we hypothesized that combination treatment may help rejuvenate the TIL population, our data did not support this hypothesis. Combination therapy tripled the PD-1 exhaustion marker score over the untreated value. This finding suggests that the therapeutic benefit of the combination treatment is mediated through a mechanism that is either nonimmunological or independent of lymphocytic exhaustion. Alternatively, PD-1 ligand may be the primary target for metformin.

Although the antitumor benefit of metformin has been demonstrated in the clinical setting, the results of clinical trials have been largely disappointing thus far [10]. The immune-potentiating benefit of metformin is well described. Several authors have demonstrated the immunostimulatory mechanism of either metformin [9–14] or metronomic cyclophosphamide [20] alone. To our knowledge, the combination regimen has not been investigated to date.

Preclinical models may provide insight into the potential applicability of drug regimens and protocols and hopefully avoid the negative outcomes of labor- and cost-intensive clinical trials.

### Limitations

In the current study, the findings in the experimental tumor model were not confirmed in models of murine orthotopic solid tumors. Lymphocyte characterization was performed with morphology alone, not with specific markers. A larger number of specimens is required to confirm the scoring results. Mechanistic studies are not covered in this research.

## Data Availability

Not applicable.

## Abbreviations

Cyclo: Cyclophosphamide
EAC: Ehrlich ascites carcinoma
H&E: Hematoxylin and Eosin
PD-1: Programmed cell Death protein − 1
TILs: Tumor-Infiltrating Lymphocytes
